# Palliative Care Admission at End‐of‐Life in Liver Cancer: A 10 Year Population‐Based Study of 3565 Deaths in Australia

**DOI:** 10.1002/cam4.71670

**Published:** 2026-02-23

**Authors:** Rebecca J. Mitchell, Shenghan Cai, Jacob George, Rose Boutros, Amany Zekry, Yvonne Zurynski

**Affiliations:** ^1^ Faculty of Medicine, Health and Human Sciences, Australian Institute of Health Innovation Macquarie University Sydney New South Wales Australia; ^2^ Storr Liver Centre The Westmead Institute for Medical Research Westmead New South Wales Australia; ^3^ Sydney Medical School The University of Sydney Camperdown New South Wales Australia; ^4^ Department of Gastroenterology and Hepatology, Westmead Hospital, Western Sydney Local Health District Westmead New South Wales Australia; ^5^ School of Clinical Medicine, St. George and Sutherland Clinical Campus University of New South Wales Kogarah New South Wales Australia; ^6^ Department of Gastroenterology and Hepatology, St George Hospital, South Eastern Sydney Local Health District Kogarah New South Wales Australia

**Keywords:** end‐of‐life‐care, hepatitis B, hepatitis C, hepatocellular carcinoma, liver cancer, palliative care

## Abstract

**Background:**

Palliative care is essential to manage symptoms and end‐of‐life (EOL) quality, yet its utilisation in liver cancer remains poorly understood. This study examined sociodemographic and clinical characteristics associated with palliative care admission in the last 5 years of life for people with a death from liver cancer.

**Methods:**

A population‐based retrospective cohort study of adults (≥ 18 years) who died from liver cancer between 2013 and 2022 in New South Wales, Australia was conducted. Multivariable logistic regression identified factors associated with palliative care admissions using linked hospital, cancer registry, notifiable conditions and mortality records.

**Results:**

There were 3565 deaths and 55.3% of people had at least one palliative care admission. Females (OR 1.25; 95% CI 1.06–1.49), individuals with anxiety‐related disorders (OR 1.63; 95% CI 1.18–2.25), drug‐related dependence (OR 1.81; 95% CI 1.29–2.54), who lived in rural locations (OR 1.39; 95% CI 1.18–1.64), who had metastatic cancer at diagnosis (OR 1.23; 95% CI 1.01–1.50), who had ≥ 4 hospital admissions (OR 1.43; 95% CI 1.08–1.90) or who died in hospital (OR 4.64; 95% CI 4.64–6.52) had a higher likelihood of a palliative care admission compared to no palliative care admissions. People admitted to intensive care (OR 0.60; 95% CI 0.47–0.75) or who had mechanical ventilation (OR 0.46; 95% CI 0.29–0.74) in the last 12 months prior to EOL were less likely to have a palliative care admission.

**Conclusion:**

This is the first Australian study to examine palliative care use in liver cancer on a population‐level. Findings support earlier integration of palliative care to reduce high‐acuity interventions. These insights have implications for service delivery, equity and policy in EOL care planning.

## 1 Introduction

Worldwide, the prevalence of liver cancer has doubled from an estimated 345,912 people in 1990 to over 739,299 people in 2021 [1]. Liver cancer often develops in the context of cirrhosis and viral hepatitis, with individuals usually presenting with advanced cancer, as the early stages of the disease are often asymptomatic [2, 3]. This limits treatment options for many individuals, resulting in a poor prognosis [2, 4, 5].

Palliative care in oncology involves providing holistic care throughout the cancer trajectory, but particularly at the end‐of‐life (EOL), to address an individual's physical, psychosocial or spiritual well‐being, while providing access to information and support to make choices about healthcare options [6, 7]. Patients with liver cancer may receive palliative care to aid relief of cancer symptoms and complications (such as pain, infection) or symptoms and complications of end‐stage liver disease (such as ascites, renal failure) [8]. However, most existing studies have focused on single‐centre cohorts or hospice‐specific datasets, limiting the generalisability of findings and underrepresenting the complexity of liver cancer care at a population‐level [9]. For example, in the United States (US), between 25% and 63% of liver cancer patients are estimated to use palliative or hospice services in the 6 months before death [2, 3]. Hospice use has been associated with lower emergency department (ED) visits, hospitalisations, intensive care unit (ICU) admissions, in‐hospital deaths [2, 3, 8] and invasive procedures [8] for liver cancer compared to not receiving hospice care.

Palliative care can play a vital role in assisting in the relief of symptoms from poor prognosis cancers [8] and in enhancing end‐of‐life‐care (EOLC) quality [6], especially when integrated into an overall management strategy [10]. The introduction of palliative care early into treatment of terminal conditions has been associated with improved patient survival and health‐related quality of life (HRQoL) [11, 12, 13]. However, patients often only receive palliative care in the advanced stages of their cancer [14]. Identifying characteristics of individuals with liver cancer who access palliative care early in their cancer trajectory, such as in the 5 years before EOL, is essential to understand real‐world practice patterns, informing equitable, system‐level models of care and will assist to guide planning for palliative care services and health system resource use. Determining whether early access to palliative care is associated with less potentially burdensome EOLC [15], may also encourage early planning for use of palliative care. This population‐based study aimed to identify sociodemographic and clinical predictors of palliative care admission in the last 5 years of life among people who died from liver cancer in New South Wales (NSW), Australia.

## 2 Methods

This is a population‐level retrospective cohort study of people who had a death from liver cancer in NSW, Australia, during 1 January 2013 to 31 December 2022. Mortality data were linked to hospital, notifiable condition and cancer registry records. An estimated 6.7 million adults aged ≥ 18 years live in NSW [16].

### 2.1 Data Sources and Linkage

Mortality data were obtained from the NSW Registry of Births, Deaths and Marriages and the cause of death unit record file (COD‐URF) and included date of death and underlying cause of death. Cause of death was classified using the International Classification of Diseases, 10th Revision (ICD‐10). The NSW Cancer Registry includes notifications of people diagnosed with cancer in NSW (excluding non‐melanoma skin cancer) and includes information on demographics, diagnosis date, cancer type and degree of spread. NSW Cancer Registry records were provided from 1997 to 2023 to identify diagnosis date and a prior history of cancer. The Notifiable Conditions Information Management System (NCIMS) contains all diagnosis notifications of certain infectious diseases, including hepatitis B (HBV) and hepatitis C (HCV), and was provided from 1997 to 2023.

Hospital records in NSW were linked for ED presentations and hospital admissions during 2007 to 2023. ED presentations to public hospitals in NSW included information on arrival and departure times, visit and separation type. Hospital admissions were to both public and private hospitals, and information included principal and additional diagnoses, clinical procedures, and separation type (e.g., hospital transfer, death). Diagnoses were classified using the International Classification of Diseases, 10th Revision Australian‐modification (ICD‐10‐AM). Procedures were classified using the Australian Classification of Health Interventions and surgical procedures (excision of lesion of liver (3041400), segmental resection of liver (3041500), lobectomy of liver (3041800), cryotherapy of liver (3041900), trisegmental resection of liver (3042100), transplantation of liver (9031700) and radiofrequency ablation of liver (5095000) were identified in the last 5 years before death). Emergency hospital admissions within 12 months of date of death were categorised as none, low (1–3 admissions) or high (≥ 4 admissions) [17]. Country of birth was identified using the Standard Australian Classification of Countries [18] in the hospital records and categorised as Australia and other countries.

The data sources were linked by the Centre for Health Record Linkage using probabilistic linkage. Upper and lower probability cut‐offs for a link were 0.75 and 0.25 and record groups with probabilities between the cut‐offs were clerically reviewed.

### 2.2 Case Inclusion Criteria

Cases included individuals aged ≥ 18 years with a liver cancer underlying cause of death (ICD‐10: C22.0–C22.9) in the COD‐URF during 2013–2022. Deaths within 30 days of liver cancer diagnosis (*n* = 911; 20.4%) were excluded due to likely advanced stage of liver cancer at diagnosis [2, 19]. The hospital service use of individuals who had an emergency presentation to the ED without admission or an emergency hospital admission and separation within 1 year of their date of death were examined.

### 2.3 Identification of Comorbidities

Both HBV and HCV notifications were identified in the NCIMS. The Charlson Comorbidity Index was used to identify 17 comorbidities using up to 50 diagnosis classifications in hospitalisation records [20]. A 5‐year lookback was applied from the date of death to identify comorbidities in the hospital admission data. Charlson comorbidities, excluding malignancies, were categorised as nil, 1 and ≥ 2 comorbidities. Comorbid conditions related to cirrhosis/liver fibrosis (ICD‐10‐AM: K74.0–K74.6), obesity (ICD‐10‐AM: E66.0–E66.2, E66.8–E66.9), alcohol misuse and dependence (ICD‐10‐AM: F10, Y90, Y91, Z50.2, Z71.4, Z72.1), drug‐related dependence (ICD‐10‐AM: F11–F16, F19, Z50.3, Z71.5, Z72.2), tobacco use (ICD‐10‐AM: F17.0–F17.9, P04.2, T65.2, Z58.7, Z71.6, Z72.0, Z81.2, Z86.43), oesophageal varices (ICD‐10‐AM: I85.0), ascites (ICD‐10‐AM: R18), hepatorenal syndrome (ICD‐10‐AM: K75.7), peritonitis (ICD‐10‐AM: K65), acute kidney injury (ICD‐10‐AM: N17.9) and mental health (ICD‐10‐AM: F10–F99), with a subset of depression (ICD‐10‐AM: F20.4, F31.3, F31.4, F31.5, F32, F33, F34.1, F41.2, F43.2) and anxiety‐related disorders (ICD‐10‐AM: F40–F48), were also identified using hospital records.

### 2.4 Palliative or Hospice Care Admission

Palliative or hospice care admissions were identified using a combination of data items in hospitalisation records that indicated palliative or hospice care (i.e., episode of care type, service‐related group, unit type on admission, peer‐group, separation mode) or an additional diagnosis in up to 50 diagnosis codes of palliative care (ICD‐10‐AM: Z51.5) [21] at least once in the 5 years preceding EOL. The first episode of palliative care during the 5 year study timeframe was identified to calculate the mean time and standard deviation (SD) between the palliative care episode and death.

### 2.5 Geographic Location and Socio‐Economic Status

The Australian Statistical Geographical Standard Remoteness Area [22] and Local Government Area (LGA) of residence in the NSW Cancer Registry or hospital records was used to derive five remoteness categories based on distance to service centres. These groupings were categorised as: urban (i.e., major cities) and rural (i.e., inner regional, outer regional, remote and very remote). Socioeconomic disadvantage was assigned using the index of relative socioeconomic disadvantage [23] and LGA of residence in the NSW Cancer Registry or hospital records. The values were partitioned into quintiles from most (i.e., 1) to least disadvantaged (i.e., 5). The quintiles are derived from Australia's population census using information including education, employment, occupation and income.

### 2.6 Data Management and Analysis

Data were analysed using SAS 9.4 (SAS Institute, Cary NC) within the Secure Unified Research Environment (SURE). All hospital episodes of care related to the same event were linked to form a period of care. Descriptive analysis described the number of ED presentations without admission and emergency hospital admissions in the last 12 months prior to death. Chi‐square tests of independence, *t*‐tests or the Wilcoxon Rank Sum Test, as appropriate, were used to examine the characteristics of individuals who had a palliative care admission in the last 5 years before EOL.

A multivariable logistic regression model was fitted to examine the association of sociodemographic and clinical characteristics associated with a palliative care admission in the 5 years before EOL. Individual predictors that were significant at *p* < 0.25 in univariate analyses were included in the multivariable model where significance was assessed at *p* < 0.05. Variables included in the final model were those available in the data and had previously been associated with mortality [2, 3, 19, 24]: sex, anxiety‐related disorder, drug‐related dependence, urban/rural residential location, cancer degree of spread at diagnosis, and emergency admission, ICU admission or mechanical ventilation in last 12 months and death in‐hospital. A Hosmer and Lemeshow Goodness‐of‐fit test [25], indicated that the model fit was adequate (*p* = 0.23).

## 3 Results

There were 3565 liver cancer deaths during 2013–2022. Of these, 1973 (55.3%) people had at least one palliative care admission in the last 5 years before EOL. During the study timeframe, patients who received palliative care were indicated to have a palliative care episode on average 50.8 days (SD 148.9, median 12 days IQR 36.0) before death. Compared to no palliative care admissions, individuals with ≥ 1 Charlson comorbidity (excluding malignancy) (82.8% vs. 90.2%), cirrhosis/liver fibrosis (39.1% vs. 42.8%), an anxiety‐related disorder (4.3% vs. 7.8%), depression (1.3% vs. 2.7%), tobacco use (61.8% vs. 67.4%), alcohol dependence (26.8% v 27.2%), drug‐related dependence (3.5% vs. 7.3%) or who lived in rural areas (25.2% vs. 29.0%) had a higher proportion of palliative care admissions (Table 1).

**TABLE 1:** Demographic characteristics by palliative care hospital admission in the last 5 years before liver cancer death.

	No palliative care admission (*n* = 1592; 44.7%)		Palliative care admission (*n* = 1973; 55.3%)		*p*
	*n*	%	*n*	%	
Mean age at death (SD)	68.5	(11.4)	68.1	(11.5)	0.4
Age group at death					
18–64	599	37.6	783	39.7	0.3
65–74	482	30.3	611	31.0	
75–84	388	24.4	425	22.1	
≥ 85	123	7.7	144	7.3	
Sex					
Male	1241	78.0	1487	75.4	0.07
Female	351	22.1	486	24.6	
Country of birth					
Australia	913	57.4	4175	59.6	0.0006
Other country	613	38.5	759	38.5	
Not known	66	4.2	39	2.0	
Number of Charlson comorbidities (excluding malignancy)^a^					
Nil	274	17.2	194	9.8	< 0.0001
1 comorbidity	471	29.6	597	30.3	
≥ 2 comorbidities	847	53.2	1182	59.9	
Other comorbidities					
Hepatitis B diagnosis (yes)	123	7.7	171	8.7	0.3
Hepatitis C diagnosis (yes)	373	23.4	500	25.3	0.2
Diabetes (yes)	521	32.7	682	34.6	0.2
Renal disease (yes)	126	7.9	136	6.9	< 0.0001
Cirrhosis/liver fibrosis (yes)	623	39.1	845	42.8	< 0.0001
Oesophageal varices (yes)	32	2.0	51	2.6	0.3
Ascites (yes)	551	34.6	788	39.9	0.001
Hepatorenal syndrome (yes)	98	6.2	96	4.0.9	0.09
Peritonitis (yes)	168	10.6	199	10.1	0.6
Acute kidney injury (yes)	597	37.5	647	32.8	0.003
Obesity (yes)	14	0.9	12	0.6	< 0.0001
Mental Health (yes)	664	41.7	973	49.3	< 0.0001
Depression (yes)	21	1.3	53	2.7	< 0.0001
Anxiety‐related disorder (yes)	68	4.3	153	7.8	< 0.0001
Tobacco use (yes)	983	61.8	1330	67.4	< 0.0001
Alcohol misuse and dependence (yes)	426	26.8	536	27.2	< 0.0001
Drug‐related dependence (yes)	56	3.5	143	7.3	< 0.0001
Geographical location of residence^b^					
Urban	1173	74.8	1398	71.0	0.01
Rural	396	25.2	570	29.0	
Socio‐economic status^b^					
Most disadvantaged	452	28.8	621	31.6	0.4
2	369	23.5	476	24.0	
3	303	19.3	361	18.3	
4	239	15.2	282	14.3	
Least disadvantaged	205	13.1	231	11.7	

^a^ Includes diabetes, mild liver disease and renal disease.

^b^ *n* = 28 not known geographic location excluded from *χ* ^2^ test of independence and *n* = 29 not known socioeconomic status excluded from *χ* ^2^ test of independence.

**FIGURE 1:**
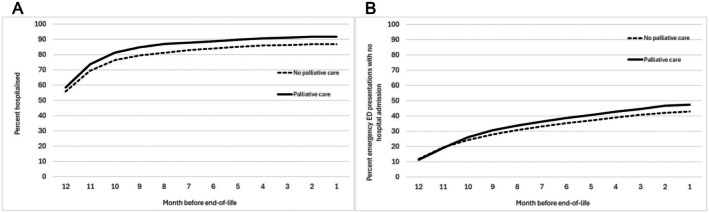
Percent with an emergency hospital admission (A) and emergency ED presentation with no admission (B) by month in last year of life by palliative care admission in last 5 years before liver cancer death.

Individuals with metastatic cancer at diagnosis (22.1% vs. 17.4%), who had ≥ 4 emergency hospital admissions in the last 12 months of life (24.6% vs. 17.2%), or who died in‐hospital (86.7% vs. 55.3%) had a higher proportion of palliative care admissions compared to no palliative care admissions. Individuals admitted to ICU (10.9% vs. 15.8%) or who received mechanical ventilation (1.8% vs. 4.2%) in the last 12 months before EOL had a lower proportion of palliative care admissions compared to none (Table 2). Individuals who had a palliative care admission had a higher proportion of emergency ED presentations without admission and emergency hospital admissions in the last 12 months of life (Figure 1).

**TABLE 2:** Cancer and clinical characteristics by palliative care hospital admission in the last 5 years before liver cancer death.

	No palliative care admission (*n* = 1592; 44.7%)		Palliative care admission (*n* = 1973; 55.3%)		*p*
	*n*	%	*n*	%	
Age at liver cancer diagnosis, mean (SD)	66.9	(11.4)	66.5	(11.2)	0.6
Age at hepatitis B diagnosis, mean (SD)	52.1	(12.7)	53.3	(13.8)	< 0.0001
Age at hepatitis C diagnosis, mean (SD)	50.9	(10.9)	49.9	(10.2)	< 0.0001
Time from liver cancer diagnosis to death (days)					
31–89	331	20.8	379	19.2	0.0008
> 90 and < 200	282	17.7	365	18.5	
≥ 200	913	57.4	1190	60.3	
Not known diagnosis date	66	4.2	39	2.0	
History of cancer (excluding liver) (yes)	76	4.8	91	4.6	0.8
Hepatocellular carcinoma cause of death (ICD‐10: C22.0)	1164	73.1	1489	75.5	0.1
Degree of cancer spread at diagnosis					
In situ/localised	692	43.5	865	43.8	0.0004
Regionalised	175	11.0	215	10.9	
Metastatic	277	17.4	435	22.1	
Not known	448	28.1	458	23.2	
Surgical procedure in last 5 years (yes)	251	15.8	282	14.3	0.2
Number of days admitted in last 12 months median (IQR)	19	(26.0)	29	(34.0)	< 0.0001
Emergency ED presentation without admission in last 12 months					
None	908	57.0	1037	52.6	< 0.0001
1–3	633	39.8	813	41.2	
≥ 4	51	3.2	123	6.2	
Emergency admission in last 12 months					
None	209	13.1	163	8.3	< 0.0001
1–3	1109	69.7	1324	67.1	
≥ 4	274	17.2	486	24.6	
ICU admission in last 12 months (yes)	251	15.8	214	10.9	< 0.0001
Mechanical ventilation in last 12 months (yes)	67	4.2	35	1.8	< 0.0001
Palliative care in last 12 months (yes)	530	33.3	1961	99.4	< 0.0001
Death in hospital (yes)	880	55.3	1711	86.7	< 0.0001
Year of death					
2013	148	9.3	161	8.2	0.6
2014	158	9.9	171	8.7	
2015	153	9.6	179	9.1	
2016	148	9.3	173	8.8	
2017	138	8.7	193	9.8	
2018	161	10.1	220	11.2	
2019	177	11.1	210	10.6	
2020	163	10.2	226	11.5	
2021	180	11.3	233	11.8	
2022	166	10.4	207	10.5	

**FIGURE 2:**
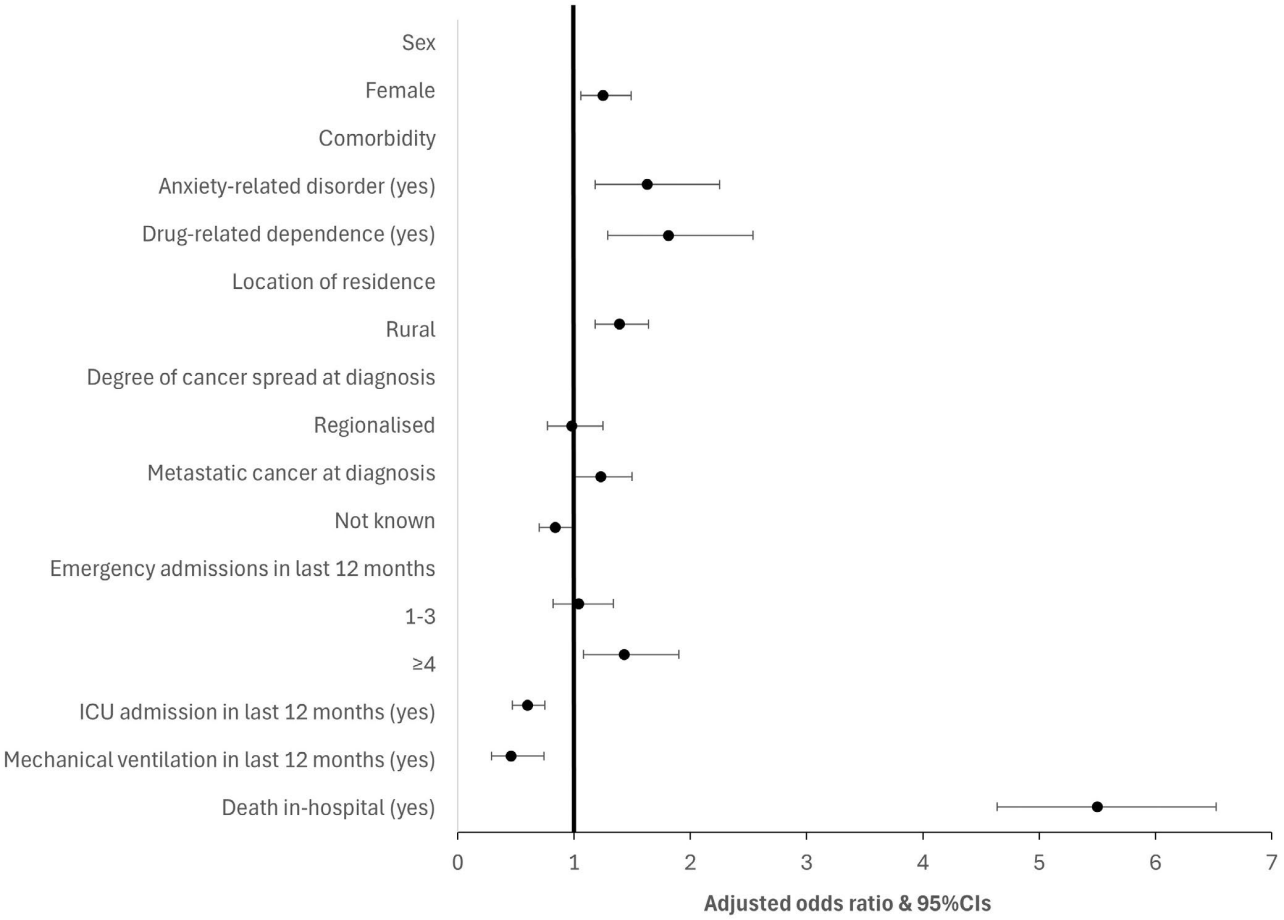
Characteristics associated with palliative care admission within 5 years of liver cancer death. Excludes *n* = 28 not known geographic location. Referent groups were: Male, no anxiety‐related disorder, no drug‐related dependence, urban location of residence, in situ/localised degree of cancer spread, no emergency admission in last 12 months, no ICU admission, no mechanical ventilation and death not in‐hospital.

Females (OR 1.25; 95% CI 1.06–1.49), individuals with anxiety‐related disorders (OR 1.63; 95% CI 1.18–2.25), drug‐related dependence (OR 1.81; 95% CI 1.29–2.54), who lived in rural locations (OR 1.39; 95% CI 1.18–1.64) or who had metastatic cancer at diagnosis (OR 1.23; 95% CI 1.01–1.50) had a higher likelihood of a palliative care admission compared to people who did not have a palliative care admission in the 5 years prior to EOL. People who had ≥ 4 hospital admissions had almost a 1.5 times higher likelihood (95% CI 1.08–1.90) and people who died in‐hospital had a five times higher likelihood (95% CI 4.64–6.52) of a palliative care admission compared to people with no palliative care admissions. People who were admitted to ICU (OR 0.60; 95% CI 0.47–0.75) or who received mechanical ventilation (OR 0.46; 95% CI 0.29–0.74) in the last 12 months of life were less likely to have a palliative care admission (Figure 2; Table S1).

## 4 Discussion

This study is the first population‐based analysis of palliative care utilisation in liver cancer in Australia, and one of the few internationally. The use of linked administrative datasets allows for a real‐world examination of the characteristics associated with palliative care admissions across diverse geographic and clinical settings in the last 5 years before EOL for people who died from liver cancer. People were more likely to have a palliative care admission if they had comorbid conditions related to anxiety or drug dependence, if they were female, lived in a rural area or had metastatic cancer at diagnosis. In terms of hospital service use, people who had ≥ 4 hospital admissions and who died in‐hospital had a higher likelihood of being admitted for palliative care, while people who had an ICU admission or received mechanical ventilation were less likely to have a palliative care admission.

As found in the current study, people with liver cancer admitted for palliative care often have a higher comorbidity burden [3, 19, 24] or a higher disease severity, as indicated by metastatic cancer at diagnosis, having ≥ 4 emergency hospital admissions or a death in‐hospital. Other studies examining use of palliative care by liver cancer patients also identified that palliative admissions are more likely for patients with advanced liver cancer [24] and that liver cancer patients with comorbidities have higher hospital use and incur higher treatment costs than patients without comorbidities [26]. People with liver cancer and a substance use disorder may have a higher likelihood of a palliative care admission due to life circumstances (e.g., lack of social support) or difficult symptom management (e.g., pain) [27]. For this cohort, getting the right mix of holistic care at EOL can be challenging [27, 28].

The current study found a slightly higher, but similar pattern of hospital service use in the last 12 months before EOL for people who had a palliative care admission compared to none. In the US, the pattern of hospital use among hospice and non‐hospice users in last 12 months of life were also similar [3] and, in Taiwan, people with liver cancer who received hospice care in the month before EOL were more likely to visit an ED or be admitted to hospital compared to no hospice care [29]. However, in Canada, in a study of patients with all types of cancer, patients who received palliative care had less ED visits and hospital admissions [30]. Liver cancer differs from all cancer types, due to both the treatment and management of cancer, and of liver failure [11]. Symptom management of cancer and liver failure is likely to be the cause of people attending an ED or being admitted to hospital in the last 12 months before EOL in the current study, particularly for people with multimorbidity, where it can be difficult to manage complex symptoms at home [6, 11, 19, 29, 31]. However, with appropriate support, cancer and liver failure symptom management at home can be achieved [32].

A palliative care admission was more likely for females and for people living in rural locations in the current study. Other studies have found conflicting results with either no difference by geographic location [19] or higher palliative care admissions for people living in urban locations at EOL [33]. This discrepancy may reflect better coordination within rural service networks in Australia or differential thresholds for hospital‐based palliative referrals. The higher rate of palliative admissions among rural residents likely reflects differences in service configuration rather than disparities in access or clinical severity. In rural NSW, hospital‐based palliative care is commonly delivered through multi‐purpose or hybrid models that integrate generalist, oncology and palliative care roles, often with lower referral thresholds [34, 35]. By contrast, metropolitan areas support specialist‐led and community‐based services that enable outpatient or home‐based palliation, thereby reducing hospital encounters. Because the data capture only inpatient activity, the rural overrepresentation may reflect coding, care and service‐delivery variation rather than actual differences in palliative need. Sex‐based differences require further investigation, particularly in the context of perceived caregiver roles or patient preferences.

Previous studies have identified that early access to palliative care for people with advanced‐stage cancers results in a better HRQoL, less potentially burdensome care at EOL, reduced symptoms of depression, reduced hospital readmissions and longer survival [6, 12, 13, 36]. People who were admitted for palliative care were less likely to have an ICU admission or receive mechanical ventilation in the last 12 months before EOL in the current study. Similarly, other studies found lower ICU admissions [2, 8, 24, 29] and less need for invasive procedures, such as intubation or ventilator support [8, 37], for people receiving palliative care. Given the association between palliative care access and lower ICU and ventilation use, often avoidable interventions, these findings support the integration of early palliative care into routine liver cancer pathways. Proactive models may reduce burdensome interventions, particularly for patients with poor prognosis or advanced disease.

Over 44% of liver cancer patients in the current cohort did not have a palliative care admission. The reasons for this are unclear. It can be challenging for clinicians to predict survival time from a liver cancer diagnosis [29] and to know the best time to seek palliative care support [11]. Patient perceptions of palliative care as only relevant at EOL can also be a potential barrier to earlier palliative care use [11, 24]. In the current study, patients had a palliative care episode a median 12 days prior to EOL. In comparison, previous Australian studies have reported a median 3 days for inpatients and 21.5 days for outpatients from a palliative care consultation to death [38], while a Malaysian study reported a median of 32 days [39]. The reasons underlying this variation in timing remain poorly understood.

For patients with end‐stage liver disease who may also be waiting a liver transplant, discussions around palliative care may not be welcome or may only occur in the days before death and focus on comfort‐based care [40]. However, advance care planning may be a mechanism to initiate conversations around using supportive, palliative care throughout the cancer trajectory [41]. For example, the Barcelona‐Clinic Liver Cancer (BCLC) protocol recommends integrated palliative care and indicates at what stage (e.g., vascular tumour invasion, lymph node or long‐distance metastasis) palliative care options should be integrated into treatment discussions with patients [42]. Hepatologists have identified additional barriers to palliative care referral including lack of training about palliative care among hepatologists, limited palliative care services, unclear referral pathways, fluctuating courses of end‐stage liver disease, unrecognised clinical triggers for palliative care, and development of complications or ascites [10, 43]. Yet, there are demonstrated benefits of palliative care referral and decreased hospital admissions and associated costs for people with end‐stage liver disease [36].

Limited community awareness of liver cancer risk, and primary prevention programs which focus predominantly on populations at‐risk of chronic HBV and HCV infection, might also contribute to late‐stage diagnoses when treatment and supportive palliative care options are limited [44, 45, 46]. For example, in the current study, of the 911 deaths within 30 days of a liver cancer diagnosis, 60.4% were of people living in the two most disadvantaged socioeconomic areas, 19.4% were diagnosed with HCV and 5.4% with HBV. Case study reports also illustrate the complexity of patient presentation with advanced liver disease that do not provide opportunity for palliative care discussions [47, 48]. In future research, deaths within 30 days of a liver cancer diagnosis should be examined to identify health and demographic characteristics of segments of the population that are not accessing timely healthcare. Furthermore, future research examining early palliative care admissions could be examined through linked health and pharmaceutical data to account for additional liver cancer and HCV/HBV therapies, including use of Lamivudine, Adefovir, Entacavir and direct acting antivirals, along with consideration of outpatient palliative care and community‐based palliative support services and their role.

Strengths of this study include that it was population‐based, utilised a 15‐year lookback period for HBV and HCV notifications and identification of a history of prior cancer. However, this study has limitations. For HBV and HCV infections to be included in the NCIMS, a person must be medically diagnosed, have a laboratory test, and any positive results must be reported by the laboratory or a clinician to their local public health unit or directly to the NICMS. This process can be influenced by changes in testing policies or screening programs over time as well as the capacity of laboratories, public health units and clinicians to notify. Hospital admissions classified as palliative care admissions were examined, but palliative care may have been provided during other hospital episodes of care and not indicated. It is possible that some palliative care episodes may have been missed being recorded and vice versa. Comorbidities were identified using hospitalisation records and comorbidities were likely under‐enumerated as comorbidities are only recorded if they influence patient treatment or care. Outpatient treatment records were not available for the time period of interest to provide information on chemotherapy or radiotherapy treatment. No information was available on prescription medications, Model For End‐Stage Liver Disease (MELD) score, Child‐Pugh scores or any patient‐reported measures (e.g., pain intensity). Data validity of hospital records was not able to be assessed.

In conclusion, liver cancer is often diagnosed at an advanced stage, making access to timely palliative care essential to improving HRQoL and reducing avoidable interventions. The study findings highlight the need for earlier identification of high‐risk groups and proactive integration of supportive care. These insights provide an evidence base to inform person‐centred models of care, reduce high‐acuity treatments near EOL, and guide policy reform in liver cancer service planning.

## Author Contributions

R.J.M. contributed to study conception and design, data analysis and original draft preparation. S.C., J.G., R.B., A.Z. and Y.Z. contributed to the interpretation of the results, provided critical feedback and approved the final version of the manuscript.

## Funding

This research was supported by the NSW Cancer Institute (2021/ATRG2028). J.G. is supported by the Robert W. Storr Bequest to the Sydney Medical Foundation, University of Sydney; a National Health and Medical Research Council of Australia Investigator and MRFF grants (APP2032407; NCRI000183; APP2016215; APP2010795; APP1196492).

## Disclosure

Artificial Intelligence Generated Content: Nil.

## Ethics Statement

Ethical approval and a waiver of consent were obtained from the NSW Population and Health Services Research Ethics Committee (2023/ETH00893). The study was conducted in accordance with relevant guidelines and regulations.

## Conflicts of Interest

The authors declare no conflicts of interest.

## Supporting information


**Table S1:** Predictors of palliative care admission within 5 years of liver cancer death.

## Data Availability

The data that support the findings of this study are available from the NSW Cancer Institute and NSW Ministry of Health. Restrictions apply to the availability of these data, which were used under licence for the current study, so are not publicly available. The research team accessed the linked data within the Secure Unified Research Environment (SURE).
