# Supplementary material for: Palliative Care Admission at End‐of‐Life in Liver Cancer: A 10 Year Population‐Based Study of 3565 Deaths in Australia
Source: Cancer Med. 2026 Feb 23;15(3):e71670. doi: 10.1002/cam4.71670 (PMC12929020; doi:10.1002/cam4.71670)
Supplement: Supplementary file 1 — Table S1: Predictors of palliative care admission within 5 years of liver cancer death. [file CAM4-15-e71670-s001.docx]

**Supplementary Table S1: Predictors of palliative care admission within 5 years of liver cancer** **death**

| **Characteristics** | **Univariate** | | **Adjusted** | |
| --- | --- | --- | --- | --- |
|  | **OR** | **95%CI** | **OR** | **95%CI** |
| **Age group at death** |  |  |  |  |
| 18-64 | 1 |  | 1 |  |
| 65-74 | 0.97 | 0.83-1.14 |  |  |
| 75-84 | 0.87 | 0.73-1.03 |  |  |
| ≥85 | 0.90 | 0.69-1.17 |  |  |
| **Sex** |  |  |  |  |
| Male | 1 |  | 1 |  |
| Female | 1.14 | 0.97-1.33 | 1.25** | 1.06-1.49 |
| **Country of birth** |  |  |  |  |
| Australia | 1 |  |  |  |
| Other country | 0.96 | 0.84-1.10 |  |  |
| Not known | 0.61 | 0.39-0.97 |  |  |
| **Number of Charlson comorbidities** (excluding malignancy)^1^ |  |  |  |  |
| Nil | 1 |  |  |  |
| 1 comorbidity | 1.67* | 1.34-2.08 |  |  |
| ≥2 comorbidities | 1.82* | 1.48-2.24 |  |  |
| **Other comorbidities** |  |  |  |  |
| Hepatitis B diagnosis (yes) | 1.15 | 0.90-1.47 |  |  |
| Hepatitis C diagnosis (yes) | 1.10 | 0.94-1.29 |  |  |
| Diabetes (yes) | 1.07 | 0.93-1.23 |  |  |
| Renal disease (yes) | 0.84 | 0.65-1.08 |  |  |
| Cirrhosis/liver fibrosis (yes) | 1.14 | 1.00-1.30 |  |  |
| Mental Health (yes) | 1.33 | 1.16-1.52 |  |  |
| Depression (yes) | 2.04** | 1.22-3.39 |  |  |
| Anxiety-related disorder (yes) | 1.86* | 1.39-2.49 | 1.63** | 1.18-2.25 |
| Tobacco use (yes) | 1.24** | 1.08-1.43 |  |  |
| Alcohol misuse and dependence (yes) | 1.00 | 0.86-1.16 |  |  |
| Drug-related dependence (yes) | 2.11* | 1.54-2.90 | 1.81** | 1.29-2.54 |
| **Geographical location of residence**^2^ |  |  |  |  |
| Urban | 1 |  | 1 |  |
| Rural | 1.21** | 1.04-1.41 | 1.39* | 1.18-1.64 |
| **Socio-economic status**^2^ |  |  |  |  |
| Most disadvantaged | 1.21 | 0.97-1.52 |  |  |
| 2 | 1.13 | 0.90-1.43 |  |  |
| 3 | 1.05 | 0.83-1.34 |  |  |
| 4 | 1.04 | 0.81-1.35 |  |  |
| Least disadvantaged | 1 |  |  |  |
| **Time from HCC diagnosis to death** (days) |  |  |  |  |
| 31-89 | 1 |  |  |  |
| >90 and <200 | 1.13 | 0.91-1.40 |  |  |
| ≥200 | 1.14 | 0.96-1.35 |  |  |
| Not known diagnosis date | 0.69 | 0.43-1.11 |  |  |
| **Degree of cancer spread at diagnosis** |  |  |  |  |
| In-situ/localised | 1 |  | 1 |  |
| Regionalised | 0.98 | 0.79-1.23 | 0.98 | 0.77-1.25 |
| Metastatic | 1.26** | 1.05-1.50 | 1.23*** | 1.01-1.50 |
| Not known | 0.85 | 0.72-1.01 | 0.84 | 0.70-1.01 |
| **Emergency admissions in last 12 months** |  |  |  |  |
| None | 1 |  | 1 |  |
| 1-3 | 1.36** | 1.09-1.71 | 1.04 | 0.82-1.34 |
| ≥4 | 2.02* | 1.56-2.62 | 1.43** | 1.08-1.90 |
| **ICU admission in last 12 months** (yes) | 0.64* | 0.53-0.78 | 0.60* | 0.47-0.75 |
| **Mechanical ventilation in last 12 months** (yes) | 0.40* | 0.26-0.61 | 0.46** | 0.29-0.74 |
| **Death in-hospital** (yes) | 5.16* | 4.37-6.08 | 5.50* | 4.64-6.52 |

^1^Includes diabetes, mild liver disease and renal disease. ^2^Excludes n=28 not known geographic location and n=29 not known socioeconomic status. *p<0.0001; **p=0.01; ***p=0.05
